# Motivations and Experiences of Volunteering Medical Students in the COVID-19 Pandemic—Results of a Survey in Germany

**DOI:** 10.3389/fpsyt.2021.768341

**Published:** 2022-01-04

**Authors:** Arndt Büssing, Alexander Lindeberg, Beate Stock-Schröer, David Martin, Christian Scheffer, Hagen S. Bachmann

**Affiliations:** ^1^Professorship Quality of Life, Spirituality and Coping, Witten/Herdecke University, Herdecke, Germany; ^2^Integrated Curriculum Anthroposophic Medicine, Anthroposophic Medicine, Witten/Herdecke University, Herdecke, Germany; ^3^Chair of Medical Theory, Integrative and Anthroposophic Medicine, Witten/Herdecke University, Herdecke, Germany; ^4^Gemeinschaftskrankenhaus Herdecke, Department for Internal Medicine, Herdecke, Germany; ^5^Chair of Pharmacology and Toxicology, Centre for Biomedical Education and Research (ZBAF), Witten/Herdecke University, Witten, Germany

**Keywords:** volunteer service, motivations to help, altruism, COVID-19, medical students, hospitals

## Abstract

**Introduction:** During the first lockdown of the COVID-19 pandemic, several medical students volunteered as assistants in hospitals, public health departments, and other healthcare services to support and substitute permanent staff. The underlying motivations to help are unclear. Therefore, we aimed to assess medical students' motivations and influencing variables such as perceived stress and burden, compassion, and indicators of spirituality.

**Materials and Methods:** Cross-sectional survey (convenience sample) from May to June 2020, directly after the first lockdown, among medical students with standardized instruments. One of them is the 12-item Motivations to Help Scale (MtHS) which was designed to fit to the population of medical students.

**Results:** Among the 731 completers, 52% were working as volunteers during the pandemic in different medical areas, most in hospitals and only a few in other areas (9% in public health departments, 6% in outpatient services), 37% would have liked to work but did not get an appropriate employment, and 21% did not intend to voluntarily support the hospital staff. Their mental burden during work was rather low, while they were somewhat affected by the personal fate of the patients. With respect to their motivations to volunteer as measured with the MtHS, *Altruistic Intentions/Helping* (Cronbach's alpha = 0.898) scored highest, followed by *Practical Application/Learning* (Cronbach's alpha = 0.808), while *Role Testing/Recognition* (Cronbach's alpha = 0.702) scored lowest. Those who volunteered had significantly higher scores for *Altruistic Intentions/Helping* and *Practical Application/Learning*, while the different phases of medical study (preclinical phase, clinical phase, and higher semester) had no influence on the extent of the students' motivation. The motivations to help were not at all or only marginally (inversely) related to indicators of stress and burden, while *Altruistic Intentions/Helping* was weakly related to affections by patients' fate.

**Conclusions:** Medical students' intention to support healthcare professionals as supplementary assistants were both prosocial and proself motivated. With this opportunity to practically apply their current knowledge and to improve their skills and competences, volunteering students might be more motivated for their further studies and their future career as compassionate medical doctors.

## Introduction

During the first phase of the COVID-19 pandemic, most hospitals had to face a relatively high number of COVID-19-infected patients that had to be treated with high amount of special care. Several of these patients were treated for several weeks in intensive care units, while others died (1). Both patients and healthcare professionals were in fear of getting infected and having a complicated course of disease (2, 3). Healthcare professionals reported high amount of compassion fatigue and burnout due to the pandemic (4), indicating an influence of the pandemic and its implications for extensive care on their mental health. Factors with a relevant impact on healthcare professionals were work overload, emotional exhaustion, risk of own infection, and transmission to their families (5), while they also had to compensate for staff shortage due to infected personnel in quarantine.

To deal with the higher amount of emotionally burdening work, retired healthcare professionals were additionally asked to support the hospitals, and nurses from other wards were trained to also care for patients with respiration requirements. While hospitals had primarily to deal with severely ill patients, other areas of the healthcare system were also critically challenged by the pandemic. Public health departments had to establish a new infrastructure for testing in a rapid and extensive way, and outpatient services had to deal with many infected patients who had to be isolated but still to be monitored to recognize a possible severe course with need for more intensive treatment. Thus, there was a shortage of healthcare staff in many areas. During that time, medical students were asked to help as supplementary assistants (6). As a response to the higher need of staff in the healthcare services, students in many countries established COVID-19 Student Response Teams (7–10)). Students interrupted their medical studies while medical schools were in lockdown, had paused clinical internships and in-class training, and were starting with online education programs (11, 12). In other cases, officials recommended that students should not be actively involved in patient care, while Miller et al. (13) clearly underlined that “in addition to the benefits to patients and the health care system, allowing students to participate reinforces important values, such as altruism, service in times of crisis, and solidarity with the profession.” In fact, several students worldwide considered supporting the healthcare services during the time of crisis (7, 13–16).

Whether and to what extent medical students were prepared, trained, and supported (also in terms of supervision while dealing with emotionally difficult situations) during this volunteer service is unclear. During their medical studies, students are confronted with large amounts of theoretical knowledge, while their practical skills and competences—particularly in terms of behaviors in pandemic situations—are rarely trained. This is particularly true for the first phases of medical study in Germany which is organized in three main phases with respective state exams: (1) preclinical knowledge transfer, (2) clinical knowledge transfer, and (3) practical training in hospitals. Most are aware that at the end of their theoretical studies, their practical skills and competences are limited. Thus, they intend to acquire and test their practical skills much earlier during their studies. The COVID-19 pandemic was such an opportunity to test theoretical knowledge and connect it with concrete service in clinical care and thus to develop new skills and competences relevant for students' medical studies and future work as medical doctors.

Therefore, we aimed to analyze medical students' motivations to help during the first phase of the pandemic. The underlying personal motivations can be diverse, and their satisfaction with their volunteering work may depend on their expectations and concrete experiences. Volunteers' motivations can be a matter of values (i.e., helping those in need to express altruistic and humanitarian values), understanding (i.e., new insights, knowledge, abilities), career (i.e., acquisition of new skills relevant for the future career), enhancement (i.e., personal growth), social (i.e., strengthening of social contacts), or protective (i.e., to reduce negative feelings and to protect the ego against difficulties of life) (17). Dolcinar and Randle (18) reduced the different motivations to three categories: “do something worthwhile, personal satisfaction, and helping others.” According to the different motivations, one could theoretically differentiate “prosocial” and “proself” persons (19). The first group would see their volunteering as an act of social responsibility (also during the pandemic), while the second would expect own benefits through their service. Both motivations could drive medical students during their mission. With respect to the prosocial motives, one would expect some of the underlying motivations to be related to medical students' compassion as an intention to generally help others. Empathic compassion as an intentional aspect of relational spirituality (20) is assumed to be an important motivator to work as a healthcare professional (21). However, there are findings that—due to different reasons—students' empathy may decrease during the course of their medical study (22). Thus, extra time to work as a volunteer could help to reconnect to their primary motivation to help and to foster and maintain their intention to compassionately care for others in need. However, stress and social support are relevant predictors of students' empathy (23). Therefore, one may assume that high stress perception during their challenging work as volunteers during the pandemic and negative experiences with COVID-19-infected patients' suffering may influence students' compassion and motivations, too. As observed in physicians and nurses, these experiences may result in emotional exhaustion and emotional distancing from the patients as a strategy, termed cooldown (24), and could influence medical students' motivations during their mission. Further, because research shows a link between volunteerism, altruism, and religiosity (25, 26), one may assume that students' underlying spirituality could also influence their motivations to help. Several religious traditions see it as a social obligation to care for persons in need (21, 27, 28), and thus, one may assume an association between spiritual issues and the motivations to help.

In this explorative study, we aimed to characterize and analyze medical students' motivations to volunteer during the first phase of the COVID-19 pandemic. As not all potentially interested medical students were employed as volunteers (due to different reasons) and as some decided against it, one would expect differences in their motivations and compassion scores. This will be addressed in this evaluation in line with putative gender differences. Because younger persons in Germany are usually not interested in specific religious issues (29, 30), we intended to address praying as an indicator of Christian religiosity, meditation as an indicator of non-religious spirituality, and perceptions of wondering awe (and subsequent feelings of gratefulness) as an indicator of perceptive spirituality relevant also for non-religious persons (31, 32). We assume that medical students' underlying spirituality is related to prosocial rather than proself motivations to volunteer during the COVID-19 pandemic. We further assume that students' motivations were negatively related to their stress perception and feelings of being under pressure during their employment. This would negatively interfere with their compassion (resulting in protective cooldown reactions) and their abilities to stop in wondering awe in specific moments.

## Materials and Methods

### Enrolled Students

In a cross-sectional open online survey, medical students from German universities were enrolled from May 29 to June 18, 2020, directly after the first lockdown. To reach as many medical students as possible, we contacted the deaneries of all German medical faculties *via* email. We requested their support to forward an email explaining our study and containing the link to the online survey to the medical students enrolled at their respective universities (using their own internal mailing lists). We further approached the German Medical Students' Association with the same request. Furthermore, we expanded the recruitment radius by posting the link to the online survey in social media (i.e., Facebook groups that were formed by medical students to organize their participation during the first wave of the COVID-19 pandemic). This study relies on a convenience sample during the first phase of the pandemic; no randomized stratified sampling was used. All students were informed about the purpose of the study, and anonymity was guaranteed; no IP addresses were recorded. By clicking the Consent button at the starting page of the survey, they were able to proceed filling the anonym German language questionnaire. Students could drop out anytime while completing the voluntary questionnaire. Further, they were able to review and change their answers. There were no incentives offered. A positive vote of the ethical committee of Witten/Herdecke University was received in May 2020 (#106/2020).

### Measures

#### Motivations to Help

As most of the available instruments to assess volunteer helping and respective functions are rather for volunteer phases prior to concrete studies or jobs, and are further not referring to the requirements of medical students who are already decided in their future perspectives, and are further less specific for the COVID-19 pandemic situation, we decided to develop a new instrument. Its main topics that should be covered refer to motives of the Volunteers Functions Inventory by Clary et al. (17). Intended topics were altruistic intentions and helping (in terms of ethical demands to help people in need, support future colleagues, and help the society in general), practical application (of what was theoretically learned during the medical studies, and increase of concrete practical knowledge referring also to the pandemic), role testing (as medical helpers), social recognition, and career. The 16 respective items of the intended Motivations to Help Scale (MtHS) to cover these topics were scored on a 7-point agreement scale ranging from *applies not at all* (1) to *applies very much* (7). The validation of this instrument will be described later on.

#### Stress Perception

Students' perceived stress was measured with the 10-item Perceived Stress Scale (PSS) (33). The internal reliability of the original PSS-10 was moderate (Cronbach's alpha = 0.78), while it was good in this study (Cronbach's alpha = 0.89). All items refer to emotions and thoughts and how often one may have felt or thought a certain way within the last days. The scores range from 1 (*never*) to 4 (*very often*); higher scores would thus indicate greater stress perception. For this study, the time frame was “the last days.”

Students were further asked whether they felt under pressure during employment. Here we differentiated time pressure, physical burden, mental burden, being concerned by lack of medical knowledge or lack of practical experience, and being affected by the personal fate of the patient. These can be scored on a 5-point Likert scale ranging (*does not apply at all*; *does not really apply*; *I don't know* (*neither yes nor no*); *applies quite well*; *definitely applies*).

#### Cool Down

Stressful situations during the job may result in emotional exhaustion and, as a strategy to cope and to remain “functional” in the job, emotional distance toward the patients (24). To measure these cooldown reactions, the 9-item Cooldown Index (CDI; Cronbach's alpha = 0.84) was used. Specific items are for example, “Patients' personal problems and worries often simply become too much for me”; “I often no longer have the patience to listen to them”; “I have to withdraw with increasing frequency to protect myself”; and “I increasingly “work to rule.”” In this study, the frequency of perceptions was scored from 1—*a few times a year or less*; 2—*once a month or less*; 3—*a few times a month*; 4—*once a week*; 5—*a few times a week*; to 6—*every day*. The scale's internal consistency was good in this sample (Cronbach's alpha = 0.83).

#### Well-Being

To assess students' well-being, the WHO-Five Well-being Index (WHO-5) was applied (34). Representative items are “I have felt cheerful and in good spirits” or “My daily life has been filled with things that interest me.” Respondents assess how often they had the respective feelings within the last 2 weeks, ranging from *at no time* (0) to *all of the times* (5). Here we report the sum scores that were referred to a 100% level [0–100], where scores <50 are indicative of reduced well-being (35). The scale's internal consistency was good in this sample (Cronbach's alpha =0.85).

#### Compassion

Compassion as perception and attitude was measured with the Santa Clara Brief Compassion Scale [SCBCS; (36)]. The five items have a very good internal congruence (Cronbach's alpha = 0.90). Specific items are “When I hear about someone (a stranger) going through a difficult time, I feel a great deal of compassion for him or her”; “I tend to feel compassion for people, even though I do not know them”; and “I often have tender feelings toward people (strangers) when they seem to be in need.” The SCBCS scores from 1 (*not at all true to me*) to 7 (*very true of me*). The scale's internal consistency was acceptable in this sample (Cronbach's alpha = 0.72).

#### Awe and Gratitude

Perceptions of wondering awe and subsequent feelings of gratitude can be regarded as an indicator of perceptive spirituality, perceived also by non-religious persons (32). To address these, we used the 7-item Awe/Gratitude (GrAw-7) scale (31). Examples of items are, “In certain places, I become very quiet and devout”; “I stop and am captivated by the beauty of nature”; “I pause and stay spellbound at the moment”; and “I stop and then think of so many things for which I'm really grateful.” The internal reliability of these items was good in the validation study (Cronbach's alpha = 0.83), and similarly good in this sample of students (Cronbach's alpha = 0.82). All items were evaluated on a 4-point scale (0—*never*; 1—*seldom*; 2—*often*; 3—*regularly*). The results were total values from 0 to 21.

#### Praying and Meditation

Frequencies of praying as a Christian form of private religious practice and meditation as a non-religious from of spiritual practice were assessed as *never, at least once per month, at least once per week*, and *at least once per day*.

### Statistical Analyses

Descriptive statistics, internal consistency (Cronbach's coefficient α), and factor analyses (principal component analysis using Varimax rotation with Kaiser's normalization) as well as first-order correlations (Spearman rho) and stepwise regression analyses were computed with SPSS 27.0. Given the exploratory character of this study, the significance level was set at *p* < 0.01. With respect to classifying the strength of the observed correlations, we regarded *r* > 0.5 as a strong correlation, an r between 0.3 and 0.5 as a moderate correlation, an r between 0.2 and 0.3 as a weak correlation, and *r* < 0.2 as negligible or no correlation.

## Results

### Enrolled Students

Among 979 students (73% women, 27% men; mean age 24 ± 4 years), 75% completed the online survey (completers) and 25% provided basis demographic data only (non-completers). Both groups did not significantly differ (data not shown). Students were recruited from all German medical universities, mainly from Hannover (19%), Aachen (15%), Gießen (9%), Münster (7%), Leipzig (8%), Berlin (5%), München (5%), and Witten/Herdecke (4%).

Among the completers [*n* = 731; 74% women, 24% men; mean age 24 ± 4 years (Table 1)], 52% were working as volunteers during the pandemic, mainly in hospitals (i.e., 33% ICU, 16% infection ward, 16% emergency unit, 16% “normal” units at hospital), and only a few in other areas (Table 1). A percentage of 52% had contact to COVID-19 infected persons (Table 1).

**Table 1:** Description of the sample of completers (*n* = 731).

	***N***	**% of responders/mean ± SD**
Gender (%)	731	100
Women	538	74
Men	188	26
Mean age	726	24 ± 4 years
Semester studying medicine	719	7 ± 3 years
Preclinical phase (1–4)	274	28
Clinical phase (5–10)	612	63
Higher semester (>10)	79	8
**Employment as volunteer (%)**	728	100
Students who were employed as volunteers	381	52
Students who were not employed as volunteers	347	48
**No employment as volunteer (%)**	347	100
Students who were not interest to be employed as volunteers	111	36
Students who were interested to volunteers but were finally not employed	183	58
Area of employment (%)*/**		
Intensive care unit	129	34
Infection unit	59	16
Emergency unit	61	16
Conventional units	60	16
Health departments	67	18
Nursing homes	0	0
Outpatient services/medical wards	21	6
Rescue ambulance	21	6
Main duties at employment (%)*	381	
Contact to COVID-19-infected patients	199	52
Care for patients	160	42
Talks with patients	121	32
Telephone contacts/support	64	17
Anamnesis	107	28
Blood draw/analysis	172	45
Monitoring of ventilation	70	18
Tracing of contact persons	65	17
**Felt under pressure during employment**		
Time pressure	381	1.5 ± 1.1 [0–4]
Physical burden	380	1.6 ± 1.2 [0–4]
Mental burden	380	1.4 ± 1.2 [0–4]
Concerned by lack of medical knowledge	381	0.8 ± 0.9 [0–4]
Concerned by lack of practical experience	380	1.1 ± 1.1 [0–4]
Affected by the personal fate of the patient	381	1.5 ± 1.2 [0–4]
**General attitudes and behaviors**		
Stress perception (PSS)	669	18.1 ± 7.6 [0–39]
Cooldown (CDI)	632	9.0 ± 7.3 [0–45]
Compassion (SCBCS)	656	1.8 ± 0.5 [0–3]
**Indicators of spirituality**		
Awe/Gratitude (GrAw-7)	656	57.1 ± 18.3 [0–100]
Praying	659	0.5 ± 0.9 [0–3]
Meditation	659	0.4 ± 0.8 [0–3]

*Option for multiple answers, and thus percentages refer to those who positively responded on the specific statement.

**Students may have volunteered in more than one setting; thus, the percentage refers to the number of students in the respective location compared to all others.

Most students were from the clinical phase of their medical study (63%), and 28% from their preclinical phase. During their employment, time pressure and physical and mental burden scored quite low, while general stress perception was in the moderate range. Cooldown perceptions were expressed low, too (Table 1). Compassion as an intention scored in the lower third.

With respect to indicators of spirituality, praying and meditation practices scored low; 15% were praying and 12% meditating at least once per week. Perceptions of Awe/Gratitude scored in the mid-range, however, with some variance (Table 1).

Most of the enrolled students were employed as volunteers (52%), while 48% were not employed as volunteers. Among those who were not employed, a large fraction was interested to volunteer but did not get a suitable employment.

### Reliability and Factorial Structure of the MtHS

In order to better deal with the different motivations to help during the Corona pandemic (Table 2), we aimed to aggregate these to comprehensible factors. A Kaiser–Meyer–Olkin value of 0.85 (as a measure for the degree of common variance) indicated that the item pool of the MtHS is suited for principal component factor analysis. Bartlett's test of sphericity was significant with < 0.0001.

**Table 2:** Reliability analysis and factorial structure of students' motivations to help.

**Motivations to help…**	**M** **[1–7]**	**SD**	**Corrected item–scale correlation**	**Cronbach's alpha if item deleted** **(alpha = 0.865)**	**Factor loading**		
					**1**	**2**	**3**
**Altruistic intentions/helping: eigenvalue 5.0, Cronbach's alpha** **=** **0.898**							
So that I can stand up for society	5.72	1.58	0.682	0.847	0.874		
When doctors and nurses are so stressed, then you have to help	5.73	1.55	0.611	0.851	0.861		
To be able to contribute something to overcoming the crisis	5.88	1.57	0.681	0.847	0.853		
When sick people are in such existential need, then you have to help	5.58	1.66	0.631	0.849	0.822		
Medical students should help deal with real health problems	5.23	1.72	0.531	0.856	0.677		
**Practical application/learning: eigenvalue 1.8, Cronbach's alpha** **=** **0.808**							
Practical application of what has been theoretically learned	4.82	1.78	0.555	0.854		0.824	
Interest in the clinical side of the pandemic	5.26	1.59	0.483	0.859		0.795	
Gaining knowledge for me	5.08	1.74	0.597	0.851		0.794	
**Role testing/recognition: eigenvalue, Cronbach's alpha** **=** **0.702**							
To get better into the medical role	3.81	1.95	0.552	0.855		0.306	0.742
To get better into the role of a helper in the healthcare sector	3.97	1.98	0.558	0.854	0.340		0.710
To get more recognition	2.51	1.60	0.268	0.871			0.689
Good for my professional qualification or CV	3.95	1.94	0.463	0.861		0.417	0.539
**Excluded items**							
Can do something to escape my own worries*	2.77	1.92					
Friends/acquaintances are also involved*	3.61	2.00					
Crediting the employment as course work**	2.66	2.12					
Financial aspects of employment**	3.71	2.15					

*Main component analysis (Varimax rotation); three factors explain 67% of variance.*

*Excluded due to a factor loading < 0.5; **deleted due to a too weak item-to-scale correlation.

During the process of validation, two personal gain items were deleted due to a too low item to scale correlation (“crediting the employment as course work,” “financial aspects of employment”), and two further items were eliminated from the item pool because of a too low factor loading (the protective motive “can do something to escape my own worries,” and the role model motive “friends/acquaintances are also involved”). An additional item addressing students' perception of whether there should be “more opportunities for such assignments” in medical studies (m16) was not included in the validation process.

Exploratory factor analysis of the remaining item pool pointed to three main components with eigenvalues > 1.0, the 5-item factor *Altruistic Intentions/Helping* with good internal consistency (Cronbach's alpha = 0.898), the 3-item factor *Practical Application/Learning* with good internal consistency (Cronbach's alpha = 0.808), and the 4-item factor *Role testing/Recognition* with acceptable internal consistence (Cronbach's alpha = 0.702). These three factors accounted for 67% of variance.

### Motivations to Help in the Sample

Students' motivation to help (as measured with the 12 item MtHS) scored highest on *Altruistic Intentions/Helping*, followed by *Practical Application/Learning*, while *Role testing/Recognition* scored lowest (Table 3). Here, we found gender-related differences in the relevance of these motivations. Further, those who were employed as volunteers had significantly higher scores for *Altruistic Intentions/Helping* and *Practical Application/Learning*, and lower stress perception, while their compassion scores were similar compared to the non-employed (Table 3). Neither students' motivations nor their compassion differed significantly between the different phases of their medical study (preclinical phase, clinical phase, and higher semester), while perceived stress was significantly higher in students from the preclinical phase of their study (Table 3).

**Table 3:** Scores of motivations to help, stress perception, and compassion in students voluntarily employed.

		**Motivation to Help (MtHS)**				
		**Altruistic intentions/helping**	**Practical application/learning**	**Role testing/recognition**	**Stress perception (PSS)**	**Compassion (SCBCS)**
All students	M	5.6	5.1	3.5	18.0	1.8
	SD	1.4	1.5	1.4	7.6	0.5
**Voluntary employment**						
Yes	M	5.9	5.3	3.6	17.3	1.8
	SD	1.1	1.4	1.3	7.2	0.5
No	M	5.3	4.8	3.5	18.8	1.9
	SD	1.6	1.5	1.4	8.0	0.5
*F*-value		40.2	16.7	1.2	6.5	1.1
*p*-value		<0.0001	<0.0001	n.s.	0.011	n.s.
**Gender**						
Women	M	5.7	5.0	3.6	18.9	1.9
	SD	1.4	1.4	1.4	7.5	0.5
Men	M	5.4	5.1	3.4	15.6	1.6
	SD	1.4	1.5	1.3	7.5	0.5
*F*-value		8.9	0.1	2.8	24.0	75.7
*p*-value		0.003	n.s.	n.s.	<0.0001	<0.0001
**Semester**						
Preclinical phase (1–4)	M	5.6	5.0	3.6	20.7	1.9
	SD	1.5	1.5	1.4	7.9	0.5
Clinical phase (5–10)	M	5.7	5.1	3.5	17.0	1.8
	SD	1.3	1.5	1.3	7.2	0.5
Higher semester (>10)	M	5.2	5.0	3.7	16.4	1.9
	SD	1.3	1.4	1.3	7.4	0.5
*F*-value		2.5	0.4	0.5	16.4	0.3
*p*-value		n.s.	n.s.	n.s.	<0.0001	n.s.

### Correlations Between Motivations to Help and Indicators of Stress and Burden

The motivations to help were not at all or marginally (inversely) only related to indicators of stress and burden (Table 4). However, there were weak positive associations between *Altruistic Intentions/Helping* and being affected by the personal fate of the patients, between the motivation of *Practical Application/Learning* and being concerned by lack of medical knowledge, and between *Role testing/Recognition* and the perception of being concerned by lack of practical experience. Students' cooldown perceptions were at least marginally negatively related to *Altruistic Intentions/Helping* and *Practical Application/Learning*, and moderately with stress perception, but not with compassion, which seems not to be affected by cooldown reactions.

**Table 4:** Correlations between motivations to help and indicators of stress/burden and compassion.

	**Altruistic intentions/helping**	**Practical application/learning**	**Role testing/recognition**	**Stress perception (PSS)**	**Compassion (SCBCS)**
**Motivations to help**
Altruistic intentions/helping	1.000				
Practical application/learning	0.342**	1.000			
Role testing/recognition	0.371**	0.444**	1.000		
Wish for more opportunities for voluntary	0.473**	0.344**	0.310**	0.026	0.116**
assignments during medical studies (m16)					
**Stress/burden indicators**					
Stress perception (PSS)	−0.026	−0.170**	0.005	1.000	0.178**
Cooldown (CDI)	−0.117**	−0.118**	0.033	0.396**	0.049
Time pressure	0.086	−0.039	−0.022	0.189**	0.026
Physical burden	0.053	0.057	−0.040	0.211**	0.090
Mental burden	0.095	0.036	0.027	0.306**	0.126
Concerned by lack of medical knowledge	0.164**	0.201**	0.185**	0.197**	0.266**
Concerned by lack of practical experience	0.141**	0.156**	0.215**	0.238**	0.271**
Affected by the personal fate of the patient	0.250**	0.178**	0.121	0.154**	0.282**
**Spirituality indicators**					
Compassion (SCBCS)	0.321**	0.090	0.147**	0.178**	1.000
Awe/Gratitude (GrAw-7)	0.161**	0.204**	0.158**	−0.122**	0.268**
Meditation	0.029	0.042	0.003	0.001	0.062
Praying	0.074	0.030	0.058	0.039	0.103**

**p <0.001 (Spearman rho); moderate correlations are highlighted (bold).

### Correlations Between Motivations to Help and Indicators of Spirituality

With respect to indicators of spirituality, *Altruistic Intentions/Helping* were moderately positively related to compassion (which is weakly related to Awe/Gratitude) and marginally only with Awe/Gratitude (which is weakly related to students' frequency of mediation and praying practice), and not at all with frequency of meditation or praying (Table 4). *Practical Application/Learning* was weakly related to Awe/Gratitude, but not with meditation or praying, while *Role testing/Recognition* was marginally only related with Compassion and Awe/Gratitude.

### Opportunities for Voluntary Assignments During Medical Studies

A large fraction of students stated that there should be “more opportunities for such assignments” in medical studies. Using a 7-grade of agreement scale, 46% agreed very much (agreement scores 6 and 7), 18% somewhat (agreement score 5), 17% were undecided, and 19% disagreed to some extent (disagreement scores 1 to 3). Interestingly, the wish to have more of such concrete learning opportunities was moderately related with all three motivation factors, particularly *Altruistic Intentions/Helping* (Table 4).

### Regression Analyses: Variables to Explain Students' Motivations to Help

As there were several variables that significantly correlated with students' motivations to help, we performed stepwise regression analysis to identify the independent variables that would best explain the variance of the dependent variable (Table 5): Gender, Compassion, Awe/Gratitude, Stress perception, Time pressure, Physical burden, Mental burden, and the perception that there should be more opportunities for such assignments in medical studies.

**Table 5:** Variables explaining best students' motivations to help (stepwise regression analyses).

		**Beta**	**T**	***p***
**Dependent variable:** ***Altruistic Intentions/Helping*** **Model 5:** ***F** **=*** **31.4**, ***p*** **<** **0.0001;** ***R*^2^** **=** **0.32**				
5	(Constant)		12.604	<0.0001
	Wish for more opportunities for voluntary assignments during medical studies (m16)	0.404	8.800	<0.0001
	Compassion (SCBCS)	0.325	6.855	<0.0001
	Mental burden	−0.128	−2.688	0.008
	Stress perception (PSS-10)	−0.139	−2.898	0.004
	Male gender	−0.104	−2.223	0.027
**Dependent variable:** ***Practical Application/Learning*** **Model 5:** ***F*** **=** **12.7**, ***p*** **<** **0.0001;** ***R*^2^** **=** **0.13**				
4	(Constant)		9.667	<0.0001
	Wish for more opportunities for voluntary assignments during medical studies (m16)	0.299	5.825	<0.0001
	Awe/Gratitude (GrAw-7)	0.137	2.685	0.008
	Stress perception (PSS-10)	−0.171	−3.204	0.001
	Mental burden	−0.122	−2.281	0.023
**Dependent variable:** ***Role testing/Recognition*** **Model 2:** ***F*** **=** **16.4**, ***p*** **<** **0.0001;** ***R*^2^** **=** **0.09**				
2	(Constant)		9.085	<0.0001
	Wish for more opportunities for voluntary assignments during medical studies (m16)	0.279	5.383	<0.0001
	Male gender	−0.105	−2.030	0.043

*In these analyses, the following independent variables were included: Gender, Compassion, Awe/Gratitude, Stress perception, Time pressure, Physical burden, Mental burden, and the perception that there should be more opportunities for such assignments in medical studies. Only variables that are significant in the explanatory models are depicted*.

*Altruistic Intentions/Helping* as a dependent variable was explained best by the wish for more of such assignments during their medical studies (explaining 17% of variance), followed by compassion (which added further 12% of explained variance), and negatively by perception of mental burden, stress perception, and male gender. Together, all five variables covered 32% of the explained variance. Time pressure, Physical burden, and Awe/Gratitude had no significant influence in this model.

*Practical Application/Learning* was explained best by the wish for more of such assignments during their medical studies (accounting for 8% of variance), followed by Awe/Gratitude, low Stress perception, and low Mental burden, which altogether would account for only 5% of further variance and are thus of minor relevance in this model. Gender, Compassion, Time pressure, and Physical burden were not among the significant variables.

*Role testing/Recognition* was explained best by students' wish for more of such assignments during their medical studies (accounting for 8% of variance), and negatively by male gender (adding only 1% of explained variance). Compassion, Awe/Gratitude, Stress perception, Time pressure, Physical burden, and Mental burden were not among the significant variables. With only 9% of explained variance, this regression model is too weak to rely on.

## Discussion

### Students' Motivations to Help

Students' motivations to volunteer in healthcare services during the first lockdown were both prosocial and proself. *Altruistic Intentions/Helping* and *Practical Application/Learning* (particularly their interest in the clinical side of the pandemic) were of strongest relevance, while *Role Testing/Recognition* as a motivation scored lowest. The factor *Altruistic Intentions/Helping* refers to prosocial motivations, while the factors *Practical Application/Learning* and *Role testing/Recognition* refer to proself motivations—although they may also have prosocial roots. This motivation pattern corresponds to the recommendation by Miller et al. that students' volunteer work during the pandemic could reinforce “important values, such as altruism, service in times of crisis, and solidarity with the profession” (13). In fact, students intended to help, to learn, and to develop as responsible persons. The most important motivations were “to contribute something to overcoming the crisis,” to “stand up for society,” and to support stressed doctors and nurses in terms of an obligation. The least important motivations were to get more social recognition or credit the voluntary service as course achievement. In accordance with our findings, “helping others, career and experiences” were relevant motivations of nursing students to help persons with mental health affections (37). During the pandemic, Tempski et al. (38) assessed “perceptions about participating in the care of patients” of medical students from Brazil. They addressed students' intention and desire to help rather than assessing those who are concretely working as volunteers as in our study. Brazilian medical students had a high sense of duty that influenced their intentions to volunteer. Nevertheless, they also stated that they are “willing to take risks by participating in practice in the context of the pandemic” (which was interpreted as an altruistic motivation). They further assumed that such a service will help them to become “a better health professional for having experienced the pandemic” (which was seen as a “perception of good performance and professional identity”) (38).

### Stress and Fear Perceptions in Students Working as Volunteers

The proself motivations found in this study did not significantly differ with respect to students' gender, while the prosocial motivations scored higher in female students. Some of the students decided against a voluntary employment in healthcare services. Those who decided against scored significantly lower on *Altruistic Intentions/Helping* and *Practical Application/Learning* than those who were employed as volunteers. However, students who decided against a voluntary employment are not less compassionate than the others but seem to be more stressed and in fear than their helping counterparts. It might be that those who decided against this volunteering assume they are not yet prepared to (theoretically and practically) deal with the medical demands. They may also fear the implications (and thus have higher stress perception) on the one hand and that they are more focused on their own concerns during their medical study (while they nevertheless do not lack compassion). Brazilian medical students who decided against volunteering were predominantly in fear of being infected themselves or felt stressed in the hospital anyway (38). Thus, both findings are in congruence at least in part. One could assume higher level of fear and stress perceptions also in German students, as most were employed in hospitals (but also in outpatient services such as public health departments, outpatient clinics) and had contact to COVID-19 infected patients (or were involved in direct care for patients and blood draw/analysis). However, students' general stress and burden scores were in the lower mid-range and their cooldown scores were rather low (and both were moderately correlated). This would indicate first that their concrete experiences in hospitals were seemingly not emotionally burdening, and second that they felt prepared for this employment. It was not a lack of knowledge that would have burdened most of them, but the time pressure and the personal fate of the patients. In fact, the underlying motivations to volunteer during the first lockdown had no relevant associations with medical students' stress perception or perceived burden during their job. There were nevertheless some exceptions: First, there was a weak correlation between *Altruistic Intentions/Helping* and the perception of being affected by the personal fate of the patients on the one hand; second, between *Practical Application/Learning* and being concerned by lack of medical knowledge; and third, between the motivation of *Role Testing/Recognition* and the perception of being concerned by lack of practical experience. These associations are sound from a theoretical point of view, because what motivates a person may also trigger the awareness of what is lacking (e.g., knowledge, practical skills, and low abilities to turn the health situation of COVID-19 infected patients). As the theoretical knowledge and practical skills may differ in the different phases of the medical study (in terms of the preclinical and clinical phase), one could expect differences in the motivations to help. However, there were no significant differences with respect to motivations or compassion scores in students from the preclinical or clinical phases or higher semester students. However, the perceived stress was significantly higher in students from the preclinical phase of their study. The later finding could be attributed to the fact that they have so far not trained their clinical skills and may have not acquired practical competences, and thus their perceived stress could be higher.

### Indicators of Spirituality and Their Influence on Students' Motivations to Volunteer

A person's religious orientation is often seen to be connected with higher motivations to altruism and compassionate care (25, 26). However, students' spiritual practices as an indicator of the centrality of these issues in their life (which scored low in the sample) had no significant association with their motivations to volunteer during the pandemic. At least their ability to stop in wondering awe (with subsequent feelings of gratitude) as an indicator of a non-religious perceptive aspect of spirituality (39) was marginally related to their motivations to volunteer. Here, the motivations for *Practical Application/Learning* were related strongest. This would indicate that medical students' spirituality is not a prominent motivator to help during the pandemic. Further, it is not necessarily a buffer of stress perception, while Awe/Gratitude may nevertheless be associated with students' compassion as an intention to help. This ability to stop in wondering awe and being grateful was found to be the best explaining variable of perceived positive changes of attitudes and behaviors during the COVID-19 pandemic (40). Medical students' perception of Awe/Gratitude is, however, better related to *Practical Application/Learning* than *Altruistic Intentions*, particularly with gaining knowledge for oneself (item m1). It might be that feelings of gratitude trigger the intention to improve the own competences and skills as a future medical doctor who compassionately cares for others in need. This may mean that in this case proself is also prosocial in terms of their future work as medical doctors.

### Implications for Students' Role in the Healthcare System

The pandemic led to a disruption of clinical education where students were not granted access to clinical learning sites. Supported active participation in the healthcare community as core of clinical learning (41) was not possible anymore. However, many students came back to healthcare services as volunteering workforces. Our results indicate that students' central motivation was to contribute to tackling the pandemic. It underlines their potential as flexible and motivated workforce willing to contribute to healthcare also before graduation. While the pandemic as an exceptional situation only allows generalizations to a limited extent, other educational projects like student-run clinics (42), students as health coaches (43), and clinical education wards (44) are further examples of students contributing to healthcare and adding value to the healthcare system (45).

Volunteering students experienced a transformation of their role in healthcare. While students in clerkship usually participate as learners without any intended impact on healthcare, volunteering students were now integrated as contributors with a meaningful role (9, 46, 47). However, as shown with our results, students are also motivated to learn when working for the healthcare service. Whether and what they learned and whether learning was supported during their helping activities should be examined in further studies.

### Limitations

This is a cross-sectional study, and thus no causal interpretations can be drawn. Due to the process of recruitment, particularly medical students with an interest to volunteer were enrolled, while it was not our main intention to also enroll students who decided against volunteering. Data should be regarded as a convenience sample and may not be representative for all students in Germany. While in 2020/2021 a total of 101,712 students were studying medicine (48), not all of them were actively engaged in the CoronAid initiative, and thus we can only report on those who participated as volunteers or intended to do so. However, a quite large group of non-volunteering students nevertheless responded to the survey, and we compared their data with the employed students. Thus, we do not assume that this group of non-employed ones is representative for all German medical students. The proportion of female medical students in this sample of volunteering students (74%) is higher as in the 2019/2020 proportion of female medical students in Germany (62%) (49). However, the proportion of volunteering younger women is lower as compared to their male counterparts (45 vs. 51% in 20–24-year-old samples, and 38 vs. 43% in 25–29-year-old samples) (50). Thus, we have a higher proportion of volunteering female students than expected from the proportion of medical students and from the background volunteering rate and proportion.

## Conclusion

During the lockdown, medical universities have stopped the education courses in presence, while several organizations encouraged medical students to volunteer in hospitals assisting their future medical colleagues and thereby improving their practical knowledge and skills. A large group of students followed this call to volunteer and were working in Intensive Care Units, Infection Units, Emergency Units, and Conventional Units. Their motivations to support healthcare professionals during the COVID-19 pandemic were mostly altruistically motivated and also to practically apply their current knowledge and to improve their skills and competences. Nevertheless, these volunteers require appropriate pre-mission training and should be working in the range of their knowledge, skills, and competences, which requires supervision by experienced medical doctors and a protective environment. In fact, it seems that the enrolled students were not too much stressed by their work, and not too much concerned by putative lack of medical knowledge and practical experience, or affected by the personal fate of the patients. This indicates that they were well-supported by the medical staff. With these new competences and skills acquired in these services, students might be more motivated for their further studies and their future career as compassionate medical doctors.

## Data Availability Statement

The raw data supporting the conclusions of this article will be made available by the authors, without undue reservation.

## Ethics Statement

The studies involving human participants were reviewed and approved by Witten/Herdecke University; ref #106/2020. Written informed consent for participation was not required for this study in accordance with the national legislation and the institutional requirements.

## Author Contributions

The CoronAid initiative encouraged this study which was finally designed by the study team. Data analysis was performed by AB and AL. The first draft of the manuscript was written by AB, HB, and CS. All authors provided feedback and approved the final manuscript.

## Conflict of Interest

The authors declare that the research was conducted in the absence of any commercial or financial relationships that could be construed as a potential conflict of interest.

## Publisher's Note

All claims expressed in this article are solely those of the authors and do not necessarily represent those of their affiliated organizations, or those of the publisher, the editors and the reviewers. Any product that may be evaluated in this article, or claim that may be made by its manufacturer, is not guaranteed or endorsed by the publisher.

## References

[1] Lai CC Wang CY Wang YH Hsueh SC Ko WC Hsueh PR. Global epidemiology of coronavirus disease 2019 (COVID-19): Disease incidence, daily cumulative index, mortality, and their association with country healthcare resources and economic status. Int J Antimicrob Agents. (2020) 55:105946. 10.1016/j.ijantimicag.2020.105946 32199877 PMC7156123

[2] Büntzel J Klein M Keinki C Walter S Büntzel J Hübner J. Oncology services in corona times: a flash interview among German cancer patients and their physicians. J Cancer Res Clin Oncol. (2020) 146:2713–5. 10.1007/s00432-020-03249-z 32415341 PMC7226710

[3] Büntzel J Micke O Klein M Büntzel J Walter S Keinki C. Take care or German Angst? Lessons from cancer care during COVID-19 pandemic in spring 2020. J Cancer Res Clin Oncol. (2021) 147:2093–105. 10.1007/s00432-020-03492-4 33387036 PMC7776312

[4] Ortega-Galán ÁM Ruiz-Fernández MD Lirola MJ Ramos-Pichardo JD Ibáñez-Masero O Cabrera-Troya J. Professional quality of life and perceived stress in health professionals before COVID-19 in Spain: primary and hospital care. Healthcare. (2020) 8:484. 10.3390/healthcare8040484 33202750 PMC7711881

[5] Shanafelt T Ripp J Trockel M. Understanding and addressing sources of anxiety among health care professionals during the COVID-19 pandemic. JAMA. (2020) 323:2133–4. 10.1001/jama.2020.5893 32259193

[6] Fischer-Fels J,. COVID-19: Aufrufe an medizinisches Personal. Deutsches Ärzteblatt vom (2020). Available online at: https://www.aerzteblatt.de/nachrichten/111190/COVID-19-Aufrufe-an-medizinisches-Personal (accessed August 13, 2021).

[7] Soled D Goel S Barry D Erfani P Joseph N Kochis M. Medical student mobilization during a crisis: lessons from a COVID-19 medical student response team. Acad Med. (2020) 95:1384–7. 10.1097/ACM.0000000000003401 32282373 PMC7188031

[8] De Moura Villela EF De Oliveira FM Toffoli Leite S Valdes RB. Student engagement in a public health initiative in response to COVID-19. Med Educ. (2020) 54:763–4. 10.1111/medu.14199 32344450 PMC7267486

[9] Haines MJ Yu ACM Ching G Kestler M. Integrating a COVID-19 volunteer response into a Year-3 md curriculum. Med Educ. (2020) 54:960–1. 10.1111/medu.14254 32438453 PMC7280601

[10] Majorski DS von Plessen CM Scheffer C Baumann H Windisch W. Coronakrise: Medizinstudierende helfen lassen. Dtsch Arztebl. (2020) 117:A–1130 / B-950. Retreived from : https://www.aerzteblatt.de/archiv/214146/Coronakrise-Medizinstudierende-helfen-lassen (accessed November 21, 2020).

[11] Sahu P. Closure of universities due to coronavirus disease 2019 (COVID-19): impact on education and mental health of students and academic staff. Cureus. (2020) 12:e7541. 10.7759/cureus.7541 32377489 PMC7198094

[12] Rose S. Medical student education in the time of COVID-19. JAMA. (2020) 323:2131–2. 10.1001/jama.2020.5227 32232420

[13] Miller DG Pierson L Doernberg S. The role of medical students during the COVID-19 pandemic. Ann Intern Med. (2020) 173:145–46. 10.7326/M20-1281 32259194 PMC7151405

[14] Gallagher TH Schleyer AM. We signed up for this!—Student and trainee responses to the Covid-19 pandemic. N Engl J Med. (2020) 82:e96. 10.1056/NEJMp2005234 32268020

[15] Valdez-Garcia JE Erana-Rojas IE Diaz Elizondo JA Cordero-Diaz MA Torres-Quintanilla A Esperon-Hernandez RI. The role of the medicine student in COVID-19 pandemic. A shared responsibility. Cir Cir. (2020) 88:399–401. 10.24875/CIRU.M20000066 32567589

[16] Hong J Jung I Park M Kim K Yeo S Lee J. Attitude of medical students about their role and social accountability in the COVID-19 pandemic. Front Psychiatry. (2021) 12:645340. 10.31234/osf.io/478ef 34140899 PMC8205150

[17] Clary EG Snyder M Ridge RD Copeland J Stukas AA Haugen J. Understanding and assessing the motivations of volunteers: A functional approach. J Pers Soc Psychol. (1998) 74:1516–30. 10.1037/0022-3514.74.6.1516 9654757

[18] Dolcinar S Randle M. What motivates which volunteers? Psychographic heterogeneity among volunteers in Australia. Voluntas. (2007) 18:135–55. 10.1007/s11266-007-9037-5

[19] De Cremer D Van Lange PAM. Why prosocials exhibit greater cooperation than proselfs: the roles of social responsibility and reciprocity. Eur J Pers. (2001) 15:5–18. 10.1002/per.41825855820

[20] Büssing A Recchia DR Dienberg T. Attitudes and behaviors related to franciscan-inspired spirituality and their associations with compassion and altruism in franciscan brothers and sisters. Religions. (2018) 9:324. 10.3390/rel9100324

[21] Thomas CL Cuceu M Tak HJ Nikolic M Jain S Christou T. Predictors of empathic compassion: do spirituality, religion, and calling matter? South Med J. (2019) 112:320–4. 10.14423/SMJ.0000000000000983 31158886

[22] Neumann M Edelhäuser F Tauschel D Fischer MR Wirtz M Woopen C. Empathy decline and its reasons: a systematic review of studies with medical students and residents. Acad Med. (2011) 86:996–1009. 10.1097/ACM.0b013e318221e615 21670661

[23] Park KH Kim DH Kim SK Yi YH Jeong JH Chae J. The relationships between empathy, stress and social support among medical students. Int J Med Educ. (2015) 6:103–8. 10.5116/ijme.55e6.0d44 26342190 PMC4561553

[24] Büssing A Falkenberg Z Schoppe C Recchia DR Lötzke D. Work stress associated cool down reactions among nurses and hospital physicians and their relation to burnout symptoms. Findings from a Cross-Sectional Study. BMC Health Serv Res. (2017) 17:551. 10.1186/s12913-017-2445-3 28797258 PMC5553651

[25] Penner LA. Dispositional and organizational influences on sustained volunteerism: An interactionist perspective. J Soc Issues. (2002) 58:447–67. 10.1111/1540-4560.00270

[26] Pawlikowski J Sak JJ Marczewski K. Physicians' religiosity and attitudes towards patients. Ann Agric Environ Med. (2012) 19:503–7. Available online at: http://www.aaem.pl/Physicians-religiosity-and-attitudes-towards-patients,71811,0,2.html 23020047

[27] Tsomo KL. Compassion, ethics, and neuroscience: neuroethics through Buddhist eyes. Sci Eng Ethics. (2012) 18:529–37. 10.1007/s11948-012-9369-4 22618161

[28] Alharbi J Al Hadid L. Towards an understanding of compassion from an Islamic perspective. J Clin Nurs. (2019) 28:1354–8. 10.1111/jocn.14725 30516863

[29] Büssing A Föller-Mancini J Heusser P. Aspects of spirituality in adolescents. Int J Children's Spirituality. (2010) 15:25–44. 10.1080/13644360903565524

[30] Büssing A Kerksieck P Föller-Mancini A Baumann K. Aspects of spirituality and ideals to help in adolescents from Christian academic high schools. Int J Children's Spirituality. (2012) 17:99–116. 10.1080/1364436X.2012.680882

[31] Büssing A Rechia DR Baumann K. Validation of the gratitude/awe questionnaire and its association with disposition of gratefulness. Religions. (2018) 9:117. 10.3390/rel9040117

[32] Büssing A Recchia DR Dienberg T Surzykiewicz J Baumann K. Awe/Gratitude as an experiential aspect of spirituality and its association to perceived positive changes during the COVID-19 pandemic. Front Psychiatry. (2021) 12:642716. 10.3389/fpsyt.2021.642716 33959049 PMC8095710

[33] Cohen S Kamarck T Mermelstein R. A global measure of perceived stress. J Health Soc Behav. (1983) 24:386–96. 10.2307/2136404 6668417

[34] Bech P Olsen LR Kjoller M Rasmussen NK. Measuring well-being rather than the absence of distress symptoms: A comparison of the SF-36 mental health subscale and the WHO-Five well-being scale. Int J Methods Psychiatr Res. (2013) 12:85–91. 10.1002/mpr.145 12830302 PMC6878541

[35] Topp CW Østergaard SD Søndergaard Bech SP. The WHO-5 well-being index: a systematic review of the literature. Psychother Psychosom. (2015) 84:167–76. 10.1159/000376585 25831962

[36] Hwang JY Plante T Lackey K. The development of the santa clara brief compassion scale: an abbreviation of sprecher and fehr's compassionate love scale. Pastoral Psychol. (2008) 56:421–8. 10.1007/s11089-008-0117-2

[37] Crawford G Burns S. Confidence and motivation to help those with a mental health problem: experiences from a study of nursing students completing mental health first aid (MHFA) training. BMC Med Educ. (2020) 20:69. 10.1186/s12909-020-1983-2 32143699 PMC7059261

[38] Tempski P Arantes-Costa FM Kobayasi R Siqueira MAM Torsani MB Amaro. Medical students' perceptions and motivations during the COVID-19 pandemic. PLoS ONE. (2021) 16:e0248627. 10.1371/journal.pone.0248627 33730091 PMC7968644

[39] Büssing A. Ehrfurcht/Dankbarkeit als säkulare Form der Spiritualität bei jungen Erwachsenen und Ordens-Christen. Spiritual Care. (2020) 9:3–11. 10.1515/spircare-2019-0057

[40] Büssing A Recchia DR Dienberg T Surzykiewicz J Baumann K. Dynamics of perceived positive changes and indicators of wellbeing within different phases of the COVID-19 pandemic. Front Psychiatry. (2021) 12:685975. 10.3389/fpsyt.2021.685975 34211412 PMC8239218

[41] Dornan T Boshuizen H King N Scherpbier A. Experience-based learning: a model linking the processes and outcomes of medical students' workplace learning. Med Educ. (2007) 41:84–91. 10.1111/j.1365-2929.2006.02652.x 17209896

[42] Schutte T Tichelaar J van Agtmael M. Learning to prescribe in a student-run clinic. Med Teach. (2016) 38:425–28. 10.3109/0142159X.2015.1072145 26313237

[43] Curry RH. Medical students as health coaches, and more: adding value to both education and patient care. Isr J Health Policy Res. (2017) 6:65. 10.1186/s13584-017-0190-z 29191229 PMC5707874

[44] Scheffer C Valk-Draad MP Tauschel D Büssing A Humbroich K Längler A. Students with an autonomous role in hospital care - patients perceptions. Med Teach. (2018) 40:944–52. 10.1080/0142159X.2017.1418504 29347873

[45] Gonzalo JD Haidet P Papp KK Wolpaw DR Moser E Wittenstein RD. Educating for the 21st-century health care system: an interdependent framework of basic, clinical, systems sciences. Acad Med. (2017) 92:35–9. 10.1097/ACM.0000000000000951 26488568

[46] Long N Wolpaw DR Boothe D Caldwell C Dillon P Gottshall L Koetter P Pooshpas P Wolpaw T Gonzalo Gonzalo JD: Contributions of Health Professions Students to Health System Needs During the COVID-19 Pandemic: Potential Strategies and Process for U.S. Medical Schools. Acad Med. (2020) 95(11):1679–1686. 10.1097/ACM.0000000000003611 32701558 PMC7375189

[47] Buckland R. Medical student volunteering during COVID-19: lessons for future interprofessional practice. J Interprof Care. (2020) 34:679–81. 10.1080/13561820.2020.1822790 32962471

[48] Rudnicka J,. Anzahl der Studierenden im Fach Humanmedizin in Deutschland nach Geschlecht in den Wintersemestern von 2007/2008 bis 2020/2021. (2021). Available online at: https://de.statista.com/statistik/daten/studie/200758/umfrage/entwicklung-der-anzahl-der-medizinstudenten/ (accessed November 15, 2021).

[49] Rudnicke J,. Studierende im Fach Humanmedizin in Deutschland nach Geschlecht bis 2019/2020. (2020). Available online at: https://de.statista.com/statistik/daten/studie/200758/umfrage/entwicklung-der-anzahl-der-medizinstudenten/ (accessed August 23, 2021).

[50] Kausmann C, Vogel, C, Hagen, C, Simonson, J,. Freiwilliges Engagement von Frauen und Männern. Genderspezifische Befunde zur Vereinbarkeit von freiwilligem Engagement, Elternschaft und Erwerbstätigkeit. Bundesministerium für Familie, Senioren, Frauen und Jugend. (2017). Available online at: https://www.bmfsfj.de/resource/blob/118460/1a128b69e46adb3fa370afc4334f08aa/freiwilliges-engagement-von-frauen-und-maennern-data.pdf (accessed August 23, 2021).

